# The O'Leary‐Sant Interstitial Cystitis Symptom Index is a clinically useful indicator of treatment outcome in patients with interstitial cystitis/bladder pain syndrome with Hunner lesions: A post hoc analysis of the Japanese phase III trial of KRP‐116D, 50% dimethyl sulfoxide solution

**DOI:** 10.1111/iju.14765

**Published:** 2021-12-20

**Authors:** Naoki Yoshimura, Takashi Uno, Mitsuru Sasaki, Akira Ohinata, Shigeki Nawata, Tomohiro Ueda

**Affiliations:** ^1^ Department of Urology University of Pittsburgh School of Medicine Pittsburgh Pennsylvania USA; ^2^ Department of Urology Ueda Clinic Kyoto Japan; ^3^ Clinical Development Center Kyorin Pharmaceutical Co., Ltd. Tokyo Japan

**Keywords:** dimethyl sulfoxide, Hunner lesion, interstitial cystitis, KRP‐116D, post hoc analysis

## Abstract

**Objectives:**

To evaluate the efficacy of intravesical KRP‐116D, 50% dimethyl sulfoxide solution, in interstitial cystitis/bladder pain syndrome patients with Hunner lesions (Hunner‐type interstitial cystitis), and to evaluate the correlations between efficacy variables and global response assessment to determine what constitutes a minimal clinically important change.

**Methods:**

We performed a post hoc analysis of the Japanese phase III trial of KRP‐116D. Changes at Week 12 from baseline in objective and subjective outcomes were compared between the KRP‐116D and placebo groups in Hunner‐type interstitial cystitis or non‐Hunner‐type interstitial cystitis patients. Correlations between efficacy variables at Week 12 and global response assessment were analyzed. Area under the receiver operating characteristic curve and the cut‐off value of efficacy valuables were calculated to determine clinically meaningful changes.

**Results:**

The effectiveness of intravesical treatment with KRP‐116D was demonstrated in Hunner‐type interstitial cystitis, but not in non‐Hunner‐type interstitial cystitis patients. Global response assessment was closely correlated with subjective outcomes including O’Leary‐Sant Interstitial Cystitis Symptom Index, O’Leary‐Sant Interstitial Cystitis Problem Index, and a numeric rating scale for bladder pain, but was less correlated with voiding variables including micturition frequency, voided volume, and maximum voided volume. In the receiver operating characteristic curve analyses, the cut‐off value for the O’Leary‐Sant Interstitial Cystitis Symptom Index was −5 (sensitivity 81.3%, specificity 83.3%).

**Conclusions:**

Clinical benefit of intravesical KRP‐116D in Hunner‐type interstitial cystitis patients was confirmed in this post hoc analysis. A five‐point reduction in O’Leary‐Sant Interstitial Cystitis Symptom Index is a clinically meaningful indicator for assessing patient satisfaction with KRP‐116D treatment in patients with Hunner‐type interstitial cystitis.

**Abbreviations & Acronyms**
BPS: bladder pain syndrome
CI: confidence interval
DMSO: dimethyl sulfoxide
FAS: full analysis set
GRA: global response assessment
IC: interstitial cystitis
ICPI: O’Leary‐Sant Interstitial Cystitis Problem Index
ICSI: O’Leary‐Sant Interstitial Cystitis Symptom Index
LS: least squares
Max: maximum
Min: minimum
MMRM: mixed model for repeated measures
NRS: numeric rating scale
ROC: receiver operating characteristic
SD: standard deviation

## Introduction

IC/BPS is a chronic bladder condition characterized by bladder pain, frequency, and urgency in the absence of other well‐defined pathologies.^1^, ^2^, ^3^ Its etiology is not well understood, nor is there an internationally accepted definition.^4^ The Japanese Guideline for Diagnosis and Treatment of IC 2007,^1^ and Clinical Guidelines for IC and Hypersensitive Bladder Syndrome 2009^5^ defined IC as a bladder pathology proven endoscopically, such as by the presence of Hunner lesions and/or mucosal bleeding after hydrodistension. However, recent reports suggest that bladder glomerulations with hydrodistension‐induced mucosal bleeding are not a specific finding of IC/BPS.^6^, ^7^ IC with Hunner lesions, the incidence of which is reportedly up to 57% in IC/BPS patients, has been reported to be clinically and pathologically different from other types of IC/BPS, and is categorized as a separate disease from non‐Hunner‐type IC/BPS.^8^, ^9^ Accordingly, in the updated Japanese^10^ and East Asian IC guidelines from 2019,^11^ IC is defined as a cystoscopy‐identified Hunner lesion phenotype only.

The clinical benefits of intravesical KRP‐116D, 50% DMSO solution versus placebo were demonstrated in a randomized placebo‐controlled double‐blind parallel phase III study,^12^ and KRP‐116D was approved for the treatment of IC patients with Hunner lesions in Japan in 2021. This was despite the fact that the KRP‐116D clinical trial included both IC patients with Hunner lesions and those with hydrodistension‐induced glomerulations according to the Japanese IC guideline from 2009. In addition, previous studies that reported the therapeutic efficacy of intravesical DMSO for relieving IC/BPS symptoms also included the bladder‐centric phenotype of IC/BPS patients identified by Hunner lesions (Hunner‐type IC) or hydrodistension‐induced glomerulations (non‐Hunner‐type IC).^13^, ^14^ Therefore, the effect of DMSO treatment has not yet been confirmed in a selected Hunner‐type IC patient population. Furthermore, a wide variety of endpoints, including ICSI, ICPI, number of micturitions per 24 h, voided volume per micturition, maximum voided volume per micturition, and an NRS for bladder pain, were used for evaluating the outcomes of clinical studies in IC/BPS. It remains to be clarified which endpoint(s) and/or how much change in an endpoint are more meaningful for predicting the minimal clinically important change.

Thus, the aims of this post‐hoc analysis of the Japanese phase III trial of KRP‐116D were as follows: (i) to evaluate the efficacy of KRP‐116D in patients with Hunner‐type IC; (ii) to evaluate the correlations between various endpoints and GRA; and (iii) to determine the cut‐off values for a minimal clinically important change in those endpoints that are closely correlated with GRA improvements.

## Methods

### Study design

This was a post hoc analysis of the randomized placebo‐controlled double‐blind study of KRP‐116D (trial registration: JapicCTI‐173566). The study was conducted at 24 sites in Japan from May 2017 to July 2018, and the study design and results have been described previously.^12^ Briefly, the study population comprised Japanese patients who exhibited the bladder‐centric phenotype of IC/BPS with Hunner lesions or hydrodistension‐induced glomerulations identified by cystoscopy and bladder‐derived pain with an ICSI of ≥9. Patients were randomly allocated to receive 50 mL of either KRP‐116D (*n* = 49) or placebo (*n* = 47). The study drug was intravesically administered every 2 weeks, six times over 12 weeks.

### Outcomes

Changes at Week 12 from baseline in ICSI, ICPI, NRS for bladder pain, number of micturitions/24 h, voided volume/micturition, and maximum voided volume/micturition were evaluated. Patients also completed a GRA questionnaire at Week 12, which is a seven‐point symmetric scale: markedly improved, moderately improved, slightly improved, no change, slightly worse, moderately worse, and markedly worse. Patients whose condition was moderately improved or markedly improved were defined as responders. The Japanese‐language versions of ICSI and ICPI, which were linguistically validated in the Japanese Guideline for Diagnosis and Treatment of IC 2007, were used.

### Statistical analysis

Post hoc analyses were performed in the FAS population, which included all enrolled patients after excluding those who did not exhibit the bladder‐centric phenotype or who had no efficacy data records after the start of treatment. Summary statistics of the change in efficacy variables at Week 12 from baseline were compared between the KRP‐116D and placebo groups in Hunner‐type IC or non‐Hunner‐type IC patients. The LS mean and two‐sided 95% CI of changes in the efficacy variables at Week 12 from baseline in each patient group were calculated using a MMRM. The difference in the LS mean and the two‐sided 95% CI between the groups was estimated using the MMRM, and the LS mean difference test (two‐sided 5% significance level) was conducted to verify the difference between the KRP‐116D and placebo groups in separate groups of patients with Hunner‐type or non‐Hunner‐type IC. Thereafter, correlation coefficients between efficacy variables at Week 12 and GRA were analyzed in all Hunner‐type IC patients pooled together regardless of their treatment status, either KRP‐116D or placebo, using Pearson’s method. For efficacy variables closely correlated with GRA, the area under the ROC curve and the cut‐off value for judgment were calculated in all Hunner‐type IC patients including those treated with KRP‐116D or placebo, together, as the value that maximizes the Youden index (sensitivity + specificity − 1).^15^

## Results

### Patients

A total of 97 patients were confirmed as having the bladder‐centric phenotype of IC/BPS identified by Hunner lesions or other pathologies such as hydrodistension‐induced glomerulations during cystoscopy. Among 96 patients in the FAS population, 86.5% (*n* = 83) had Hunner‐type IC (42 in the KRP‐116D group and 41 in the placebo group), and 13.5% (*n* = 13) had non‐Hunner‐type IC (seven in the KRP‐116D group and six in the placebo group; Table 1). Baseline demographics and disease characteristics were well balanced between the KRP‐116D and placebo groups of patients with Hunner‐type or non‐Hunner‐type IC.

**Table 1:** Summary of demographic and other baseline characteristics

	Hunner‐type IC (*n* = 83)		Non‐Hunner‐type IC (*n* = 13)	
	KRP‐116D (*n* = 42)	Placebo (*n* = 41)	KRP‐116D (*n* = 7)	Placebo (*n* = 6)
Sex, *n* (%)				
Male	5 (11.9)	2 (4.9)	1 (14.3)	1 (16.7)
Female	37 (88.1)	39 (95.1)	6 (85.7)	5 (83.7)
Age				
<65 years, *n* (%)	17 (40.5)	16 (39.0)	4 (57.1)	3 (50.0)
≥65 years, *n* (%)	25 (59.5)	25 (61.0)	3 (42.9)	3 (50.0)
Mean ± SD, years	64.9 ± 13.8	65.5 ± 13.0	55.6 ± 14.9	57.3 ± 16.1
Min, median, max, years	31, 68.5, 87	36, 67.0, 87	30, 61.0, 70	36, 61.0, 77
Weight				
<50 kg, *n* (%)	17 (40.5)	18 (43.9)	3 (42.9)	1 (16.7)
≥50 kg, *n* (%)	25 (59.5)	23 (56.1)	4 (57.1)	5 (83.3)
Mean ± SD, kg	55.40 ± 10.93	52.52 ± 12.11	58.24 ± 16.99	55.33 ± 6.28
Min, median, max, kg	38.6, 53.50, 80.5	29.6, 52.00, 86.8	42.3, 53.60, 90.0	46.5, 54.95, 63.8
Body mass index				
<25.0 kg/m^2^, *n* (%)	32 (76.2)	33 (80.5)	5 (71.4)	6 (100.0)
≥25.0 kg/m^2^, *n* (%)	10 (23.8)	8 (19.5)	2 (28.6)	0 (0.0)
Mean ± SD, kg/m^2^	23.14 ± 3.88	22.17 ± 4.41	22.70 ± 4.06	21.72 ± 2.14
Min, median, max, kg/m^2^	18.1, 22.82, 34.9	14.5, 21.39, 36.0	17.6, 23.26, 27.9	19.0, 21.73, 24.1
Type of IC, *n* (%)				
Hunner‐type	42 (100.0)	41 (100.0)	0 (0.0)	0 (0.0)
Non‐Hunner‐type	0 (0.0)	0 (0.0)	7 (100.0)	6 (100.0)
History of hydrodistension, *n* (%)				
No	6 (14.3)	8 (19.5)	0 (0.0)	1 (16.7)
Yes	36 (85.7)	33 (80.5)	7 (100.0)	5 (83.3)
History of intravesical DMSO, *n* (%)				
No	36 (85.7)	36 (87.8)	7 (100.0)	6 (100.0)
Yes	6 (14.3)	5 (12.2)	0 (0.0)	0 (0.0)
Use of drugs for primary disease at screening, *n* (%)				
No	7 (16.7)	15 (36.6)	2 (28.6)	1 (16.7)
Yes	35 (83.3)	26 (63.4)	5 (71.4)	5 (83.3)
ICSI score				
9≤, <13, *n* (%)	14 (33.3)	15 (36.6)	3 (42.9)	2 (33.3)
13≤, <17, *n* (%)	18 (42.9)	16 (39.0)	2 (28.6)	3 (50.0)
17≤, ≤20, *n* (%)	10 (23.8)	10 (24.4)	2 (28.6)	1 (16.7)
Mean ± SD	13.9 ± 3.2	13.8 ± 3.2	13.7 ± 3.1	13.2 ± 2.7
Min, median, max	9, 14.0, 20	9, 13.0, 20	11, 13.0, 19	9, 13.0, 17
ICPI score				
Mean ± SD	12.0 ± 3.1	11.9 ± 2.8	11.9 ± 4.2	11.3 ± 4.2
Min, median, max	4, 13.0, 16	6, 12.0, 16	7, 12.0, 16	6, 11.5, 16
Number of micturitions per 24 h				
Mean ± SD	15.67 ± 7.85	15.43 ± 5.92	20.86 ± 16.13	11.50 ± 1.95
Min, median, max	8.0, 14.50, 50.0	8.0, 13.50, 37.0	9.0, 16.50, 56.5	9.0, 11.75, 14.0
Voided volume per micturition, mL				
Mean ± SD	113.8 ± 52.9	110.5 ± 58.0	83.3 ± 63.0	138.6 ± 61.2
Min, median, max	27, 109.9, 257	31, 103.6, 305	38, 66.2, 217	80, 125.9, 248
Maximum voided volume per micturition				
<150 mL, *n* (%)	15 (35.7)	18 (43.9)	4 (57.1)	1 (16.7)
≥150 mL, *n* (%)	27 (64.3)	23 (56.1)	3 (42.9)	5 (83.3)
Mean ± SD, mL	183.5 ± 101.3	172.4 ± 100.9	183.9 ± 129.7	265.8 ± 94.5
Min, median, max	50, 187.5, 600	45, 160.0, 500	90, 135.0, 465	125, 257.5, 415
NRS for bladder pain				
<7, *n* (%)	25 (59.5)	26 (63.4)	5 (71.4)	4 (66.7)
≥7, *n* (%)	17 (40.5)	15 (36.6)	2 (28.6)	2 (33.3)
Mean ± SD	6.64 ± 1.46	6.54 ± 1.56	5.62 ± 1.21	6.28 ± 1.10
Min, median, max	4.0, 6.33, 10.0	4.0, 6.67, 10.0	4.3, 5.00, 7.0	5.0, 6.17, 8.0

Analysis set: FAS.

### Efficacy in Hunner‐type IC patients

The change in the LS mean ICSI at Week 12 from baseline in Hunner‐type IC patients was −5.6 in the KRP‐116D group and −3.4 in the placebo group. The estimated difference between the KRP‐116D and placebo groups was −2.2 (95% CI −3.8, −0.6; *P* = 0.0080 [Table 2]). Similar efficacies were shown in LS mean changes from baseline in ICPI and NRS for bladder pain at Week 12 and estimated differences between KRP‐116D and placebo. The LS mean change at Week 12 from baseline, and estimated differences between KRP‐116D and placebo in voiding variables including number of micturitions/24 h, voided volume/micturition (mL), and maximum voided volume/micturition (mL) were also statistically significant. The proportions of responders on GRA at Week 12 in the KRP‐116D and placebo groups were 53.7% (22/41) and 27.5% (11/40), respectively. The difference in the proportion of responders between the KRP‐116D and placebo groups was 26.2% (95% CI 4.8, 44.4; *P* = 0.0236).

**Table 2:** Changes in efficacy variables from baseline to Week 12

	Hunner‐type IC		Non‐Hunner‐type IC	
	KRP‐116D (*n* = 42)	Placebo (*n* = 41)	KRP‐116D (*n* = 7)	Placebo (*n* = 6)
ICSI score				
No. of subjects at Week 12	41	39	7	6
LS mean change at Week 12 from baseline	−5.6	−3.4	−2.9	−3.8
(95% CI)	(−6.7, −4.4)	(−4.5, −2.2)	(−5.4, −0.3)	(−6.5, −1.1)
Estimated difference	−2.2		0.9	
(95% CI for estimated difference)	(−3.8, −0.6)		(−2.8, 4.7)	
*P* value	0.0080		0.6008	
ICPI score				
No. of subjects at Week 12	41	39	7	6
LS mean change at Week 12 from baseline	−5.3	−2.2	−2.2	−3.6
(95% CI)	(−6.4, −4.2)	(−3.3, −1.1)	(−5.4, 1.0)	(−7.0, −0.1)
Estimated difference	−3.1		1.3	
(95% CI for estimated difference)	(−4.7, −1.5)		(−3.4, 6.0)	
*P* value	0.0002		0.5467	
Number of micturitions per 24 h				
No. of subjects at Week 12	41	39	7	6
LS mean change at Week 12 from baseline	−3.86	−1.27	−3.06	−5.76
(95% CI)	(−5.01, −2.72)	(−2.44, −0.10)	(−4.99, −1.14)	(−7.85, −3.67)
Estimated difference	−2.59		2.70	
(95% CI for estimated difference)	(−4.23, −0.96)		(−0.26, 5.66)	
*P* value	0.0023		0.0688	
Voided volume per micturition (mL)				
No. of subjects at Week 12	41	39	7	6
LS mean change at Week 12 from baseline	45.2	11.9	−5.1	35.3
(95% CI)	(32.6, 57.9)	(−0.9, 24.7)	(−26.6, 16.4)	(11.9, 58.7)
Estimated difference	33.4		−40.4	
(95% CI for estimated difference)	(15.4, 51.4)		(−73.3, −7.5)	
*P* value	0.0004		0.0203	
Maximum voided volume per micturition (mL)				
No. of subjects at Week 12	41	39	7	6
LS mean change at Week 12 from baseline	52.3	7.1	−9.5	24.0
(95% CI)	(36.0, 68.5)	(−9.3, 23.5)	(−54.9, 35.9)	(−25.2, 73.1)
Estimated difference	45.2		−33.4	
(95% CI for estimated difference)	(22.1, 68.2)		(−101.6, 34.8)	
*P* value	0.0002		0.3048	
NRS for bladder pain				
No. of subjects at Week 12	41	39	7	6
LS mean change at Week 12 from baseline	−3.28	−2.05	−0.54	−2.26
(95% CI)	(−3.99, −2.57)	(−2.77, −1.34)	(−1.91, 0.82)	(−3.73, −0.78)
Estimated difference	−1.23		1.72	
(95% CI of estimated difference)	(−2.23, −0.22)		(−0.31, 3.74)	
*P* value	0.0173		0.0896	
GRA				
No. of subjects at Week 12	41	40	7	6
Responder	22 (53.7)	11 (27.5)	3 (42.9)	3 (50.0)
Non‐responder	19 (46.3)	29 (72.5)	4 (57.1)	3 (50.0)
Difference	26.2		−7.1	
(95% CI)	(4.8, 44.4)		(−48.5, 37.6)	
*P* value	0.0236		1.0000	

Analysis set: FAS.

### Efficacy in non‐Hunner‐type IC patients

In non‐Hunner‐type IC patients, no statistical difference was found between the KRP‐116D and placebo groups in LS mean changes at Week 12 from baseline in ICSI, ICPI, NRS for bladder pain, or voiding variables including number of micturitions/24 h and maximum voided volume/micturition (Table 2). The proportions of responders on GRA at Week 12 in the KRP‐116D and placebo groups were 42.9% (3/7) and 50.0% (3/6), respectively. In addition, the statistical power of the *t*‐test was not higher than 0.438 for any of the study endpoints due to the small number of non‐Hunner‐type IC patients in this study (KRP‐116D, *n* = 7; placebo, *n* = 6 [data not shown]).

### Correlations between efficacy variables and GRA, and ROC curves in patients with Hunner‐type IC

**Fig. 1:**
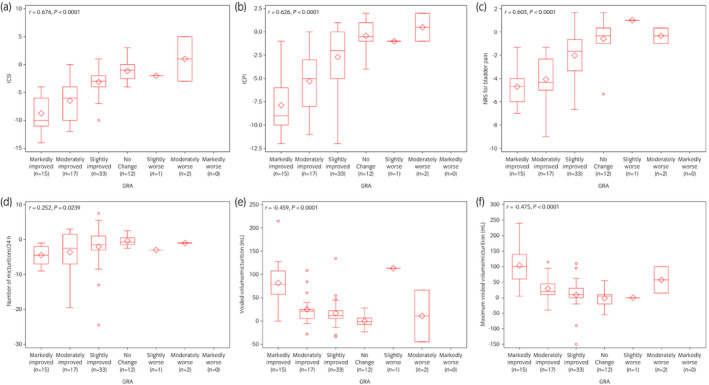
Correlations between GRA and (a) ICSI, (b) ICPI, (c) NRS for bladder pain, (d) number of micturitions/24 h, (e) voided volume/micturition (mL), and (f) maximum voided volume/micturition (mL) at Week 12 after intravesical treatment with KRP‐116D or placebo. Each figure shows boxplot. *r*, correlation coefficient.

**Fig. 2:**
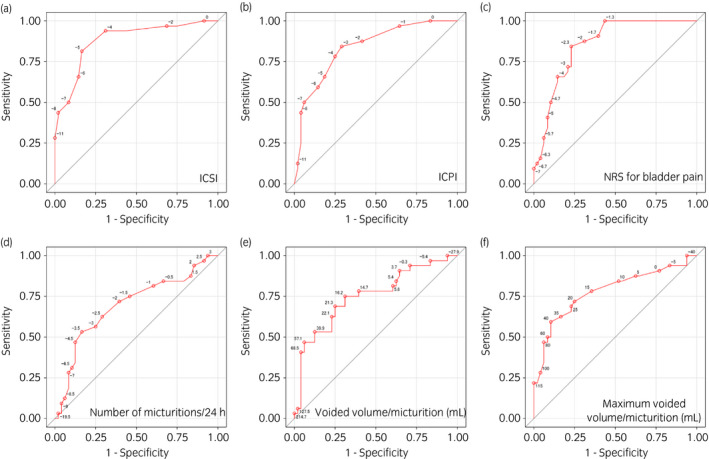
ROC curves for (a) ICSI, (b) ICPI, (c) NRS for bladder pain, (d) number of micturitions/24 h, (e) voided volume/micturition (mL), and (f) maximum voided volume/micturition (mL) at Week 12 after intravesical treatment with KRP‐116D or placebo. The areas under the curves for ICSI, ICPI, NRS for bladder pain, number of micturitions/24 h, voided volume/micturition (mL), and maximum voided volume/micturition (mL) were 0.8789, 0.8350, 0.8532, 0.6947, 0.7484, and 0.7793, respectively.

At Week 12 after intravesical treatment with KRP‐116D or placebo, Pearson correlation coefficients for GRA with ICSI, ICPI, and NRS for bladder pain were 0.676, 0.626, and 0.603, respectively; however, the Pearson correlation coefficients for voiding variables such as micturitions/24 h, voided volume/micturition (mL), and maximum voided volume/micturition (mL) were lower at 0.252, −0459, and −0.475, respectively (Fig. 1). Next, ROC curves for ICSI, ICPI and NRS for bladder pain were constructed (Fig. 2). The areas under the curve for ICSI, ICPI and NRS for bladder pain were 0.8789, 0.8350, and 0.8532, respectively. The cut‐off values for clinically meaningful differences in ICSI, ICPI, and NRS for bladder pain were −5 (sensitivity 81.3%, specificity 83.3%), −3 (sensitivity 84.4%, specificity 70.8%), and −2.3 (sensitivity 84.4%, specificity 77.1%), respectively.

## Discussion

In the randomized placebo‐controlled double‐blind phase III study of KRP‐116D, it was shown that there were clinical benefits for patients with the bladder‐centric phenotype of IC/BPS including both Hunner‐ and non‐Hunner‐type IC, which were defined according to the 2009 Japanese IC guideline^1^ available at the time when the study was designed. In the updated 2019 guidelines,^10^ a new definition of IC/BPS was introduced, in which only Hunner‐type IC is defined as IC while other types are categorized as BPS. Thus, in this post hoc analysis of the phase III study of KRP‐116D, we performed separate evaluations for the effectiveness of KRP‐116D in patients with Hunner‐type IC, who represented 86.5% of enrolled patients, and those with non‐Hunner‐type IC with hydrodistension‐induced glomerulations. As a result, better clinical outcomes were demonstrated in Hunner‐type IC patients than in non‐Hunner‐type IC patients for subjective endpoints including ICSI, ICPI, and NRS for bladder pain, as well as for the objective endpoints including number of micturitions/24 h, voided volume/micturition, maximum voided volume/micturition. The proportion of responders to KRP‐116D on GRA was 53.7%, compared to 27.5% with placebo in patients with Hunner‐type IC. In contrast, KRP‐116D was not found to be effective in patients with non‐Hunner‐type IC. This is in line with the findings of a previous study by Tomoe^16^ showing that the Hunner‐type IC patient is a good responder to DMSO compared to the non‐Hunner‐type IC patient. However, it is also possible in our KRP‐116D trial that the number of non‐Hunner‐type IC patients (*n* = 7 and *n* = 6 in the respective groups) was too small to provide adequate power for a meaningful statistical comparison between the placebo and KRP‐116D groups. Previous studies have shown that non‐Hunner‐type IC/BPS patients have limited inflammatory changes in the bladder wall and that the bladder epithelium is usually well preserved, and inflammatory cell infiltration in the lamina propria of non‐Hunner‐type IC/BPS bladders is much less remarkable compared to that seen in the Hunner‐type IC bladder.^17^ However, a recent study reported that Hunner‐type IC bladders and a proportion of non‐Hunner‐type IC bladders with severe (grade 3) glomerulations exhibited similarities in terms of decreased urothelial cytoskeleton and cell proliferation protein expression in the bladder epithelium, suggesting a common pathophysiological process in Hunner‐type IC and a subgroup of non‐Hunner‐type IC.^18^ Furthermore, neovascularization with overexpression of angiogenic factors such as platelet‐derived endothelial cell growth factor or vascular endothelial growth factor in the bladder mucosa has been identified as an important mechanism leading to glomerulations in non‐Hunner‐type IC/BPS.^19^, ^20^, ^21^ Therefore, further phenotyping based on mucosal pathologies including angiogenic changes would be necessary to identify those bladder‐centric IC patients suitable for intravesical DMSO treatment in the non‐Hunner‐type IC population.

Moderate correlations between GRA and IC symptom variables including ICSI, ICPI, and NRS for bladder pain were observed. Among them, the correlation coefficient of GRA with ICSI was the highest (*r* = 0.676). However, the correlations between GRA and voiding variables were limited, with coefficient values of lower than 0.5. In particular, the reduction in micturition frequency showed the lowest coefficient value (0.252), suggesting that it was not sufficient for an improvement in GRA. In our studies, the micturitions/24 h were significantly reduced by 12‐week treatment with KRP‐116D from 16.4 to 12.4 in bladder‐centric IC/BPS patients^13^ and from 15.7 to 11.9 in Hunner‐type IC patients (this study) although micturition frequency was still high after KRP‐116D treatment. The reason for this disconnection between GRA and micturition frequency after KRP‐116D treatment in Hunner‐type IC patients is not known. However, a previous study reported that, in IC/BPS patients treated with conventional therapies, although pain was improved after 3 months, micturition frequency was not improved in more than half of patients.^22^ It could therefore be speculated that the reduction in micturition frequency after KRP‐116D treatment was not enough to be perceived as a satisfactory improvement in GRA. Also, the possibility that some IC/BPS patients still chose to urinate frequently as a learned form of prophylactic behavior despite their improvement in pain sensation cannot be excluded. Further long‐term observational studies are needed to clarify these points. If a satisfactory reduction in micturition frequency is achieved by a new generation of treatments other than DMSO, GRA could be further improved in IC/BPS patients.

In the ROC curves, the five‐point or higher change in ICSI is a clinically useful cut‐off value for predicting responders to KRP‐116D with higher specificity (83.3%) compared to ICPI (70.8%) and NRS (77.1%). In accordance with this, Lubeck *et al*.^23^ reported that a −5.38 change in ICSI was a clinically meaningful difference using patients’ Overall Rating of Improvement of Symptoms in a randomized, double‐blind clinical study of pentosan polysulfate sodium in patients with IC/BPS. These results as well as our own suggest that a reduction of five points in ICSI can be used as an adequate indicator for predicting a favorable treatment outcome in real‐world clinical settings.

This study has some limitations. First, the number of patients with non‐Hunner‐type IC was too small to draw a conclusion regarding the effectiveness of KRP‐116D in those patients. Further studies are therefore needed that include a larger number of non‐Hunner‐type IC patients to investigate efficacy in this patient population. Second, most IC/BPS patients need to receive lifetime treatment; however, the treatment period included in this study was only 12 weeks. Additional clinical studies with a long‐term treatment duration are needed.

In conclusion, the clinical benefits of intravesical KRP‐116D in Hunner‐type IC patients was confirmed in this post hoc analysis of the phase III study. A five‐point reduction in ICSI was found to be a clinically meaningful indicator for assessing patient satisfaction with KRP‐116D treatment in Hunner‐type IC patients.

## Author contributions

Naoki Yoshimura: Conceptualization; Methodology; Supervision; Writing – original draft; Writing – review and editing. Takashi Uno: Conceptualization; Methodology; Writing – review and editing. Mitsuru Sasaki: Conceptualization; Methodology; Writing – review and editing. Akira Ohinata: Conceptualization; Methodology; Writing – review and editing. Shigeki Nawata: Conceptualization; Formal analysis; Methodology; Writing – review and editing. Tomohiro Ueda: Conceptualization; Methodology; Supervision; Writing – review and editing.

## Conflict of interest

N. Yoshimura and T. Ueda report personal fees from Kyorin, during the conduct of the study. Outside the submitted work, N. Yoshimura reports personal fees from Kyorin and Astellas, T. Ueda reports personal fees from Kyorin, Seikagaku and Japan Tobacco. T. Uno, M. Sasaki, A. Ohinata and S. Nawata are employees of Kyorin Pharmaceutical Co., Ltd.

## Approval of the research protocol by an Institutional Reviewer Board

The study protocol of the Japanese Phase III trial of KRP‐116D were approved by the institutional review board at each participating study site (KRP116D‐L301).

## Informed consent

All patients gave written informed consent before initiation of any study‐specific procedures.

## Registry and Registration No. of the study/trial

JapicCTI‐173566.

## Animal studies

N/A.
